# CCND2 and miR-206 as potential biomarkers in the clinical diagnosis of thyroid carcinoma by fine-needle aspiration cytology

**DOI:** 10.1186/s12957-023-02899-w

**Published:** 2023-01-24

**Authors:** Shifa Yuan, Zhijun Liu, Shanshan Yu, Xiaolei Wang, Jian Shi

**Affiliations:** 1 Department of General Surgery, Hospital of Hebei Province Crop of Chinese Armed Police Force, Shijiazhuang, Hebei China; 2 Department of Otolaryngology, the Fourth Hospital of Hebei Medical University, Shijiazhuang, 050000 Hebei China

**Keywords:** Thyroid carcinoma, Fine-needle aspiration cytology, miR-206, Cyclin D2

## Abstract

**Background:**

To investigate the relationship between cyclin D2 (CCND2) and miR-206 expression in fine-needle aspiration cytology of thyroid carcinoma.

**Methods:**

A total of 65 patients with thyroid carcinoma were selected as the subjects and 65 patients with benign thyroid nodules were in control group. The fine-needle aspiration cytology of thyroid nodules was performed. CCND2 and miR-206 levels were detected by PCR.

**Results:**

Compared with the patients with benign thyroid nodules, the expression level of miR-206 in fine-needle aspiration cytology of thyroid cancer patients decreased significantly and the expression level of CCND2 increased significantly. CCND2 and miR-206 expression was negatively correlated in thyroid cancer tissues. Area under curve (AUC) of miR-206 level in the diagnosis of thyroid cancer was 0.889, and the sensitivity and specificity were 92.3% and 81.5%, respectively. AUC of CCND2 level in the diagnosis of thyroid cancer was 0.837, and the sensitivity and specificity were 67.7% and 89.2%, respectively. The AUC of combined detection of CCND2 and miR-206 in the diagnosis of thyroid cancer was 0.959, and the sensitivity and specificity were 93.8% and 87.7%, respectively. The levels of miR-206 and CCND2 were significantly correlated with TNM staging and lymph node metastasis.

**Conclusions:**

miR-206 and CCND2 may become new biomarkers for clinical diagnosis of thyroid cancer based on the fine-needle aspiration cytology of thyroid nodules.

## Background

Thyroid carcinoma is a tumor of the endocrine system. With the increasing awareness of physical examination, thyroid nodules have been examined frequently [1, 2]. In clinical diagnosis of thyroid nodules, the thyroid fine-needle aspiration method is commonly used. This method has the advantages of high safety, simple operation and low cost, and can accurately determine the nature of thyroid nodules, timely detect thyroid malignant tumors, and avoid surgical treatment for benign thyroid patients [3–5]. Numerous studies showed that microRNAs (miRNAs) and human cyclin D2 (CCND2) are implicated in tumorigenesis [6–8]. Di Fiore et al. [9] found that the let-7d miRNA reduced the proliferation of osteosarcoma cells by downregulating CCND2 and upregulating p21 and p27 cyclin-dependent kinase (CDK) inhibitors. However, at present, there are no reports on the correlation of miR-206 and CCND2 in FNAC of thyroid cancer. Therefore, this study aimed to investigate miR-206 and CCND2 levels in fine-needle aspiration cytology (FNAC) of patients with thyroid cancer and those with benign thyroid nodules and explored the relationship between CCND2 and miR-206 in FNAC for the potential in clinical diagnosis of thyroid cancer.

## Materials and methods

### Patients

This study was approved by Ethics Committee of Hebei Medical University (Approval No. 35421), and all patients provided informed consent. The samples were collected from the patients with FNAC of thyroid nodule at Fourth Hospital of Hebei Medical University from January 2019 to December 2020. Thyroid ultrasound revealed that all patients had thyroid nodules that met the thyroid fine-needle aspiration indications. After pathological diagnosis, 65 patients with thyroid carcinoma were selected as the subjects, including 28 men and 37 women; aged from 35 to 65 years old, with an average age of 51.22 ± 5.49 years old. Thirty-three cases had lymph node metastasis, while 32 cases had no lymph node metastasis. TNM staging was based on AJCC (American Joint Committee on Cancer) TNM system [10], including 35 cases in stage I and 30 cases in stages II–III. For tumor size: ≤ 4 cm in 37 cases, and > 4 cm in 28 cases. Based on histological type of thyroid carcinomas, 52 patients had papillary thyroid cancer, 6 had follicular thyroid cancer, and 7 had other types of thyroid cancer. In addition, 65 patients with benign thyroid nodules (histological type was follicular adenoma) were selected as the control group, including 36 males and 29 females, aged from 33 to 62 years old, with an average age of 49.37 ± 5.68 years old. There was no significant difference (*P* > 0.05) in gender and age between the two groups of patients.

Diagnostic criteria for thyroid cancer and benign thyroid nodules were based on the guidelines published previously [11]. The inclusion criteria are as follows: (1) thyroid nodules with suspicious metastatic lymph nodes in the neck; (2) the patients with complete clinical data; (3) all patients were not treated with any antitumor therapy before operation, and the patients had clear pathological diagnosis after operation. The exclusion criteria are as follows: (1) recent use of anticoagulants; (2) the patients with other organ diseases were intolerant to fine-needle aspiration; (3) female patients with menstruation to avoid possible interference of hormones.

Diagnostic criteria for thyroid fine-needle aspiration were based on the guidelines of the Papanicolaou Society of Cytopathology, and divided into six types: (1) undiagnosed: there is a problem of sampling, so the cytological diagnosis cannot be carried out; (2) benign lesions: non-neoplastic diseases; (3) follicular lesions: tend to be benign lesions, but the possibility of tumors is not ruled out; (4) follicular tumors: adenoma or carcinoma; (5) suspected malignancy: have malignant cytological features, and undiagnosed; (6) malignant tumors [12].

### Specimen collection

Ultrasound was used to localize the thyroid lesions. After local anesthesia, the patient was subjected to fine-needle aspiration under ultrasound guidance. Two fine-needle aspiration tissues from 65 patients with thyroid carcinoma and 65 patients with benign thyroid nodules were collected. One of them was sent for pathological examination, and the other one was stored in liquid nitrogen for PCR analysis.

### PCR

The total RNA was isolated from tissue samples using Trizol reagent (Sigma-Aldrich, USA), and used for reverse transcription into cDNA using PrimeScript RT kit (Synbio Technologies, Suzhou, China). PCR was performed using SYBR Premix Ex Taq kit (Synbio Technologies, Suzhou, China), and the primers for miR-206, CCND2, GAPDH, and U6 which were synthesized by Sangon Biotech (Shanghai, China). The primer sequences are shown in Table 1. The reaction procedure is as follows: 95 °C for 20 s and 40 cycles of 95 °C for 10 s, 60 °C for 20 s, 72 °C for 10 s. U6 and GAPDH were used as internal reference for miR-206 and CCND2, respectively, and the expression levels of miR-206 and CCND2 were calculated by 2^−ΔΔCt^ method.

**Table 1:** Primer sequences

Gene	Forward primer 5′-3′	Reverse primer 5′-3′
miR-206	TGACAAAGGCAG-GAGGTA	ATCTCTGGGTGCTGG-TGAAGG
CCND2	GCAGAAGTGCGAAGAGGAGG	GCTTGATGGAGTTGTCGGTGTA
GAPDH	GAAGGTGAAGGTCGGAGTC	AAGATGGTGATGGGATTTC
U6	AAGGTGAAGGTCGGAGTCAAC	GGGGTCATTGATGGCAACAATA

### Statistical analysis

Data were presented as mean ± standard deviation (*x̅* ± *s*) and analyzed by SPSS 17 software. The *T* test was used to compare two groups, and one-way ANOVA (analysis of variance) was used to compare multiple groups. The Pearson correlation was used for the analysis of the correlation between CCND2 and miR-206. The ROC curve was used to analyze the values of miR-206 and CCND2 for the diagnosis of thyroid cancer. *P* < 0.05 was considered significant.

## Results

### miR-206 and CCND2 expression in FNAC of thyroid cancer

Compared with the patients with benign thyroid nodules, miR-206 level in FNAC of thyroid cancer patients decreased significantly (P<0.05), while CCND2 level increased significantly (*P* < 0.05) (Table 2).

**Table 2:** Expression levels of miR-206 and CCND2 in fine-needle aspiration cytology of patients with thyroid carcinoma and benign thyroid nodules

Group	*n*	miR-206	CCND2
Patients with benign thyroid nodules	65	4.15 ± 1.63	1.12 ± 0.59
Patients with thyroid carcinoma	65	1.38 ± 0.93	2.52 ± 0.93
*t* value	*-*	19.060	41.197
*P* value	*-*	0.000	0.000

*t* value was calculated by *t*-test

### The correlation between CCND2 and miR-206 in FNAC of thyroid cancer patients

**Fig. 1:**
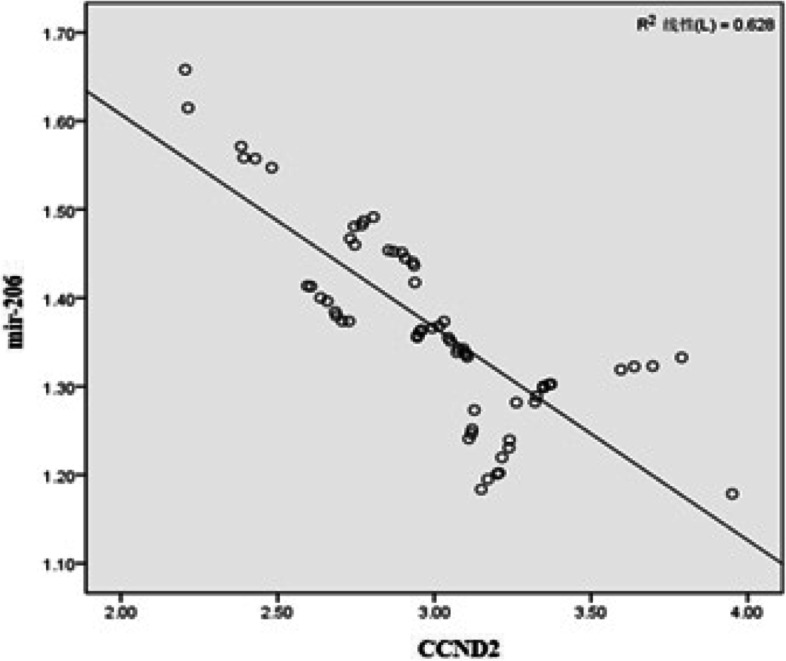
Correlation between CCND2 and miR-206 levels in fine-needle aspiration cytology of thyroid cancer patients

By Pearson correlation analysis, we found negative correlation between CCND2 and miR-206 levels in FNAC of thyroid cancer patients (*r* = − 0.693, *P* < 0.05) (Fig. 1).

### CCND2 and miR-206 levels in FNAC for the diagnosis of thyroid cancer

**Fig. 2:**
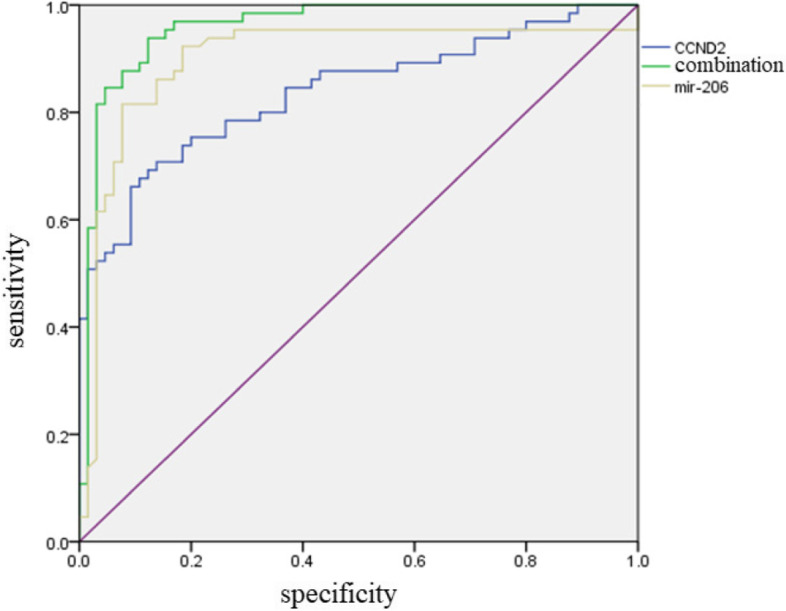
The values of CCND2 and miR-206 levels in the diagnosis of thyroid cancer by ROC curve analysis

Based on ROC curve analysis, the area under curve (AUC) of miR-206 level for the diagnosis of thyroid cancer was 0.889 (95% confidence interval (CI): 0.836–0.963), the cut-off value was 2.967, and its sensitivity and specificity was 92.3% and 81.5%, respectively. The AUC of CCND2 level for the diagnosis of thyroid cancer was 0.837 (95% CI: 0.768–0.907), the cut-off value was 1.985, and its sensitivity and specificity was 67.7% and 89.2%, respectively. The AUC of combined detection of miR-206 and CCND2 levels for the diagnosis of thyroid cancer was 0.959 (95% CI: 0.924–0.993), and its sensitivity and specificity was 93.8% and 87.7%, respectively (Fig. 2).

### The relationship between CCND2 and miR-206 levels in FNAC and the clinicopathological parameters of thyroid cancer patients

CCND2 and miR-206 levels in FNAC were significantly related to TNM staging and lymph node metastasis (*P* < 0.05) but not to tumor size in thyroid cancer patients (*P* > 0.05) (Tables 3 and 4).

**Table 3:** Relationship between miR-206 and clinicopathological parameters of thyroid cancer patients

Parameters		*n*	miR-206	*t* value	*P* value
Lymph node metastasis	Yes	33	1.16 ± 0.87	1.913	0.046
	No	32	1.59 ± 0.96		
TNM staging	Stage I	35	1.66 ± 0.93	2.424	0.018
	Stages II–III	30	1.11 ± 0.89		
Tumor size	≤ 4 cm	37	1.31 ± 0.91	0.433	0.667
	> 4 cm	28	1.41 ± 0.94		

*t* value was calculated by *t*-test

**Table 4:** Relationship between CCND2 and clinicopathological parameters of thyroid cancer patients

Parameters		*n*	CCND2	*t* value	*P* value
Lymph node metastasis	Yes	33	2.98 ± 0.95	3.545	0.001
	No	32	2.17 ± 0.89		
TNM staging	Stage I	35	3.01 ± 0.95	3.677	0.000
	Stages II–III	30	2.07 ± 0.91		
Tumor size	≤ 4 cm	37	2.58 ± 0.93	0.390	0.698
	> 4 cm	28	2.49 ± 0.91		

*t* value was calculated by *t*-test

## Discussion

The incidence of thyroid cancer has increased recently, and early diagnosis and therapy of thyroid cancer is particularly important in the clinic. miRNA is single-stranded RNA molecule with a length of 20–23 nucleotides (nt) and is an important regulator of gene expression, including the genes that mediate tumorigenesis [13]. Recent studies suggest that miRNAs could be novel biomarkers for cancer [14, 15].

CCND2 is a member of cyclin family and plays a pivotal role in controlling cell cycle transition from G1 phase to S phase. Eisfeld et al. found that CCND2 could increase the phosphorylation of retinoblastoma protein, promote cell cycle progression and cell proliferation, thereby promoting tumorigenesis [16]. Takano et al. found that CCND2 overexpression was implicated in gastric cancer [17].

Up to now, miR-206 has been implicated in a variety of cancers such as gastric cancer, colorectal cancer, and lung cancer, and miR-206 mainly inhibits tumorigenesis by targeting oncogenes [18–20]. Interesting, a study showed that miR-206 participated in thyroid hormone regulation [21]. To our knowledge, only one study reported the involvement of miR-206 in thyroid cancer, and it was shown that miR-206 was significantly downregulated in papillary thyroid carcinoma [22]. Consistent with previous reports, in this study, we found that miR-206 level in FNAC of thyroid cancer patients decreased significantly, while CCND2 level increased significantly, compared to the patients with benign thyroid nodules. These results indicate that the abnormal expression of miR206 and CCND2 may be closely associated with the occurrence and development of thyroid cancer.

The Pearson correlation analysis showed a negative correlation of CCND2 and miR-206 levels in FNAC of thyroid cancer patients. CCND2 could be the direct target of miR-206 and miR-206 inhibits cell cycle progression in thyroid carcinoma by downregulating the expression of CCND2. ROC curve analysis indicated that single or combined analysis of CCND2 and miR-206 had diagnostic value for thyroid cancer, and the diagnostic value of combined detection was higher. Furthermore, CCND2 and miR-206 levels were not significantly correlated with the tumor size of thyroid cancer patients but were correlated with lymph node metastasis, indicating that miR-206 and CCND2 may be involved in tumor metastasis. Further studies are necessary to determine whether miR-206 and CCND2 can be integrated into the nomogram to improve the accuracy of predicting lymph node metastasis of thyroid cancer [23, 24]. However, it should be noted that more than one miRNA is necessary to improve the accuracy of disease diagnosis based on miRNA markers. In this aspect, Mazeh et al. developed a diagnostic panel of 19 miRNAs to differentiate benign from malignant thyroid nodules in FNAC [25].

This study has several limitations. First, this is a single-center study. Second, the sample size is relatively small. Third, functional studies are necessary to elucidate the mechanisms by which miR-206 and CCND2 are negatively correlated and regulate the metastasis of thyroid cancer. Large-scale multiple-center prospective studies are necessary to validate our conclusion.

In summary, we found that miR-206 expression was low and CCND2expression was high in FNAC of thyroid cancer patients, and the expression of CCND2 and miR-206 was negatively correlated. CCND2 and miR-206 may be new biomarkers for clinical diagnosis of thyroid cancer.

## Data Availability

All data are available upon request to the correspondence author.
